# A description of classroom help networks, individual network position, and their associations with academic achievement

**DOI:** 10.1371/journal.pone.0208173

**Published:** 2018-12-19

**Authors:** Louise Gerharda Maria van Rijsewijk, Beau Oldenburg, Tom Augustinus Benedictus Snijders, Jan Kornelis Dijkstra, René Veenstra

**Affiliations:** 1 Department of Sociology, University of Groningen, Groningen, the Netherlands; 2 Inter-university Center for Social Science Theory and Methodology (ICS), Utrecht, the Netherlands; 3 Nuffield College, University of Oxford, Oxford, United Kingdom; Middlesex University, UNITED KINGDOM

## Abstract

This study examined how classroom peer relations can be described in terms of the network of help relations among students, and the positions students take up in this help network, and whether the structure of adolescent classroom help networks and individual network positions were associated with academic achievement. Help networks were based on the peer nomination question "Who helps you with problems?" Building on previous studies on classroom climate and individual network position, higher academic achievement was expected in classrooms with: a dense help network; no or a few network isolates (referring to students that did not give or receive help at all); less segmentation in help relations; equally distributed help nominations. In addition, higher achievement was expected for individuals with more helpers and a more central position in the help network. Using the Dutch SNARE data (54 classrooms; 1,144 students), the multilevel models suggested that lower achievement was related to an unequal distribution of help relations in a classroom. Moreover, the centrality of individuals in the help network was linked to higher achievement. Classrooms varied strongly on network dimensions, and networks that would theoretically be expected to be most beneficial for achievement (with high density, a few isolates, low segmentation, and high equality) turned out to be highly uncommon. The findings demonstrated that subtle network processes were relevant for academic success, and that classroom network characteristics are associated with classroom-level variation in academic achievement. Descriptive results underlined the complexity of the social context of classrooms, and the absence of 'beneficial' classrooms suggests that researchers should adjust their notion of what is a beneficial or detrimental classroom environment for adolescents.

## Introduction

Adolescents spend a large portion of their days in classrooms in the presence of their classmates. For this reason, it is important to know how students in the classroom get along with each other, and how these peer relations affect students’ adjustment. Researchers have acknowledged this importance, and found that a positive classroom social climate has beneficial effects on many outcomes, including academic adjustment, mental health, and socio-emotional functioning [1, 2].

Traditionally, the classroom social climate has been captured using student perceptions of, amongst others, the extent to which classmates are nice toward each other [3]. In more recent years, social network researchers added to this by capturing the social climate more explicitly, demonstrating which students have positive relations with whom, and how these relations together shape the overall classroom climate. Using this social network perspective, it has been found that students in classrooms with a centralized network structure (referring to a network in which students vary strongly in their number of social relations) are more supportive of aggressive behaviors [4], less behaviorally and academically engaged [5], and more likely to develop psychological problems at later age [6].

Notwithstanding the importance of investigating the effect of classroom social climate on adjustment, one’s individual position in the classroom network of social relations is as important for adjustment [7]. What is more–the overall classroom social climate is constructed from the social relations individuals have with their classmates. Therefore, when investigating individual adjustment in the school context, research on the effects of peer relations should not focus on classroom social climate and individual social position as independent constructs, but should examine these in concert.

In addition, there is need to further examine how characteristics of the classroom network structure coincide within and vary over classrooms. Researchers argued that social network information can be utilized to initiate or stimulate change in behaviors or relations [8, 9]. For example, social network data may provide information about whether all students are connected in the network, or whether links between students can be altered in order to make the network more cohesive. Therefore, it is important to gain knowledge on how classroom social networks can be characterized, and how individuals are embedded in these networks.

Furthermore, research on positive relations in classrooms has focused mainly on ‘liking’ relations, best friendships, and ‘hanging out together’. So far, no study has examined help networks and the individual position in help networks, which is a significant aspect of the classroom social climate.

This study focuses explicitly on how classrooms can be described in terms of the network of help relations among students, and the positions individuals take up in this help network. Because we study these help relations in the school context, we are interested in how the structure of the help network and one’s position in this network affect an outcome that is salient to the school context: academic achievement. In this way, we build on previous research on classroom social climate by assessing the association of classroom network characteristics on academic achievement in conjunction with the effects of individual network position. Moreover, we extend previous work by taking a closer look at the characteristics of help networks and the individual embeddedness in these networks. This study may provide insights into whether help networks can be used as a basis for teachers to assess where in the classroom network they may intervene to improve the overall classroom social climate and students’ network position, which may impact their academic success.

## Theoretical background

### Classroom help network

To clarify what is meant by a ‘help network’ and ‘help relations’, we now explain shortly how the help network is measured. We identified the adolescent network of helpers using a so-called peer nomination technique. Peer nominations have been frequently used to identify relations or interactions between individuals—for example, friendships, liking, and also help [10, 11]. Following this procedure, we asked adolescents to identify classmates who ‘*help them with problems (for example*, *with homework*, *with repairing a flat [bicycle] tire*, *or when you are feeling down)*?’ Aggregating these help relations to the classroom level, global network patterns can be distinguished. We focused on *cohesion* within the help network, *segmentation* of help relations, and *inequality* in the number of help relations. Cohesion refers to the extent to which help relations in the classroom are present; segmentation refers to the tendency of students to limit their (help) interactions to a select group of classmates; and inequality refers to an unequal division of support relations in the classroom, in which some students have many helpers and others no or a few helpers. Together, these dimensions capture not only the presence of help relations, but also the way in which help relations are patterned.

#### Cohesion

As environment for academic and socio-emotional development, it is argued that classrooms may function as ‘competence enhancing contexts’, or ‘optimal learning environments’, stimulating students’ engagement in academic activities [12]. Optimal classroom climates are described as environments in which students are connected with each other through positive, supportive relationships. In such contexts, students respect and trust each other, and feel safe and valued by peers, providing a good foundation for academic learning. Particularly classrooms with cohesive help networks may function as competence enhancing contexts, because the widespread giving and receiving of help is highly reflective of such a foundation [13, 14]. Asking for help requires trust toward peers, and the confidence that one will not be rejected or ridiculed as a response. Helping others requires the capacity to put oneself in others’ position and the ability to respectfully concern their issues. These positive characteristics may affect students’ motivation to go to school and to participate in academic activities by making their classroom a safe and enjoyable place [15]. Students are less likely to skip school when students respect, trust, and care about others [16], and a general positive school climate stimulates the completion of homework and active participation in classroom academic activities [17]. Other studies found that being in peer contexts characterized by positive and supportive relations is related to less individual learning difficulties [18], and that students show higher academic motivation when they expect each other to share and cooperate [19].

Second, students are likely more able to focus on school work when they feel emotionally and physically healthy [20]. Although researchers did not focus explicitly on help, it was found that positive classroom climates contribute to student health outcomes [2]. For example, the extent to which students perceive their schoolmates to like and befriend each other has been associated with less symptoms of depression [21]. In addition, students show more emotional problems in classrooms where they witnessed negative peer interactions or marginalization of other classmates, even if they were not marginalized themselves [22, 23]. Similarly, negative peer climates were found to predict psychosomatic complaints, such as headaches and stomachaches, trouble falling asleep, and loss of appetite [24]. Arguably, these complaints might affect the concentration and ability to complete schoolwork and participate actively in the classroom. Therefore, we expect that cohesion in the help network is positively associated with academic achievement (Hypothesis 1).

#### Segmentation

Not only *whether* students help others may matter, it may also matter whether students limit their helping interactions to a specific set of peers. Part of what has been previously defined as a negative classroom atmosphere is ‘“the extent to which students refuse to mix with the rest of the class” [25]. Students might help others, but limiting it to a small group of familiar classmates. Whereas such a pattern does not necessarily mean that one refuses to mix with other classmates, helping classmates outside of the boundaries of one’s group might indicate more generalized respect and trust. In line with this, it was found that children show better academic adjustment in classrooms where children do not limit their play interactions to a specific set of peers [26], and school grades were lower in classrooms where children ‘hung out’ with each other in cliques [27]. Based on this, we expect that segmentation of the help network is negatively associated with academic achievement (Hypothesis 2).

#### Inequality

Previous research has indicated that inequality in the division of social relations is salient for student academic outcomes: Students were more engaged in academic tasks in classrooms that had equally distributed ‘hanging out’ relations, and highly equal networks buffered the negative effects of student difficulties (e.g., behavioral and relational problems) on student academic engagement [5]. Inequality may also elicit or increase emotional symptoms [6, 28, 29] and stimulate the approval of aggressive behavior in the classroom [4, 30, 31], both which might negatively affect achievement. The underlying mechanism explaining these findings might be that inequality in the division of social relations might trigger social comparison and competition between classmates [4]. Inequality in help implies unequal access to the social (e.g., affection) and instrumental (e.g., access to knowledge and skills) benefits that help provides. This might be especially detrimental for adolescents, who generally develop a heightened concern for their position in the peer group [32–34]. Research pointing in this direction demonstrated that competition in the school context increases adolescents’ academic self-consciousness, indicating that students were fearful to make mistakes in front of classmates, were embarrassed in school, and nervous to perform in front of peers [35], potentially hampering their school adjustment. Taken together, we expect that inequality in the help network is negatively associated with academic achievement (Hypothesis 3).

### Individual position in the help network

Whereas a general abundance of help relations in the classroom may foster academic achievement by providing a pleasant learning atmosphere, students own relations with classmates matter as well [7]. Generally, the social position of students in classrooms has been assessed by asking students whether they felt accepted and valued by classmates [36–38], or by using peer nominations on (dis)liking and friendship, and constructing labels such as ‘popular’, ‘rejected’, and ‘neglected’ [39–41]. The present study follows a similar peer nomination approach. However, we determine the network position of students by: the number of times they nominate classmates as helpers; whether they are isolated from the network (referring to that they do not receive and give nominations for help); the centrality of their location in the network (referring to whether they can easily ‘access’ peers in the help network). We chose these indicators of individual network position as they have parallels in the classroom network structure indices. Cohesion has a direct parallel in the individual number of helpers and the individual being an isolate. Segmentation and inequality are network concepts that are not direct aggregates of individual network positions. To have a richer image of the individual position in the classroom network, we do not only take into account the number of helpers and isolation, but also their centrality, referring to their social distance to other classmates.

#### Number of helpers, isolation, and centrality

Generally, previous research has shown that individual perceptions of classroom belonging (e.g., whether peers wanted to work with or liked the individual) affect academic motivation and expectancies for academic success in early adolescents [36]. Comparable findings have been reported for the perception of being valued and respected by peers [37], the perception of having supportive classmates [42], and being included in the peer group [38, 43]. Moreover, academic outcomes vary among students with different peer status, with high-status students generally having higher achievement than their low-status peers [40, 41]. Furthermore, ‘invisibility’ in the classroom (having neither negative nor positive peer and teacher relations) was related to relatively low school liking [44] and low achievement [39, 43].

Similar findings arguably apply to help: Help by classmates offers social benefits and may hence foster classroom belonging. Additionally, the informational and instrumental benefits provided by help may help students to tackle (academic) problems and improve achievement. Receiving help from classmates has been related to increased academic motivation in early and middle adolescents [19]. In addition, a central position in the help network might aid to find potential help(ers): When one is helped by a few classmates, or only by a specific group of classmates, access to resources and the diversity of resources might be limited. In line with this, it has been found that university students performed better if they sought advice from a large number of peers in the network, but also if their social distance to all others in the advice network was shorter [45]. Similarly, adolescents who indicated they ‘hung out’ with multiple peer cliques in the classroom showed higher academic achievement [43]. Thus, we expect that: the number of helpers mentioned by students is positively associated with academic achievement (Hypothesis 4); isolation from the help network is negatively associated with academic achievement (Hypothesis 5); higher individual centrality in the help network is positively associated with academic achievement (Hypothesis 6).

#### The present study

We aim to assess whether variation in the characteristics of help networks and variation in individual embeddedness in these networks is related to adolescents’ academic outcomes. Previous findings have established that adolescents’ academic motivation and success are in part determined by the social climate in the classroom, of which peer support is a salient aspect. The present study provides more insight into what help networks look like and how they may be associated with adolescents' school achievement. We expect higher achievement in classrooms with a cohesive help network; in classrooms where help relations are less segmented; and in classrooms with equally distributed help relations. In addition, we expect higher achievement for individuals with more helpers and a more central position in the help network.

## Methods

### Procedure

We use data from the SNARE-project (Social Network Analysis of Risk behavior in Early adolescence [46]), a study aimed at investigating the co-evolution of social networks and social development among adolescents. Prior to the data collection, all eligible students and their parents received an information letter, in which they were asked to participate. If students wished to refrain from participation, or if their parents disagreed with their children’s participation, they were requested to send a reply card or email within ten days. We emphasized during every assessment that participation was anonymous and could be terminated at any point in time. The SNARE study has been approved by the ethical committee of one of the participating universities. During the assessments, a teacher and research assistant(s) were present. The research assistant gave a brief introduction, and the students then filled in the questionnaire on the computer during class. The assessment of the questionnaires took place during regular school hours within approximately 45 minutes. The students who were absent that day were assessed within a month.

### Participants

We examined the networks of all first- and second year classrooms of one participating school in the north of the Netherlands. For this study, we used the networks as assessed in December 2011 to examine associations with academic achievement as assessed in April 2012. Students who joined or left the school during the period December–April were removed from the sample, as for the joiners all network data were missing, and for leavers school grades were missing. For five classrooms, no school grades were available. The study sample contained 54 participating classrooms (*M* classroom size = 21.27 students, *SD* = 4.69) and 1,144 students (*M* age = 13.11 years, *SD* = 0.66, 49.0% boys, 94.7% Dutch). Students had, on average, a slightly lower SES than the average Dutch SES. In total, 35 students were absent during the assessment in December, and 39 students were absent during the assessment in April. However, this affected only the information on their individual network position. Furthermore, some participants (30 students across 20 classrooms) named (almost) everyone in their classroom as helper, whereas they hardly named anyone at the preceding and/or next assessment. In addition, their help nominations were hardly or not reciprocated (whereas earlier research has found that 45%-49% of the help nominations were mutual [47]). These extreme (out)degree outliers may have interpreted the question differently from their classmates. Also, they distorted the structure of the classroom network. We recoded their outgoing nominations as missing, but all other information about these outliers (their incoming nominations, classroom network indices, and achievement) was retained. Previous network research has used a similar strategy to handle extreme outdegree outliers [48].

### Measures

**Academic achievement.** Information on students’ academic achievement was retrieved from the school administration (derived from the school report card), presenting the average of grades received until April for each subject separately. Grades can range from 1 (lowest) to 10 (highest), where 5.5 or higher stands for a pass. We calculated the average grade over three school subjects of which the final exam is compulsory for students in every academic track: Dutch language, English language, and mathematics.

**Classroom help network.** The structure of the classroom help network and individual network position were calculated on the basis of a peer nomination question, for which students were asked to name classmates who ‘*help you with problems (for example*, *with homework*, *with repairing a flat [bicycle] tire*, *or when you are feeling down)*?’ A similar peer nomination question was used in previous studies investigating adolescent help relations, where they were associated with inter- and intraethnic relationships [10] and popularity [11].

**Cohesion: Density and proportion of isolates.** Cohesion in the help network was captured using two indices: Density, and the proportion of isolates. The density of a network refers to the number of relations in the network relative to the possible number of relations in the network (if everyone were to nominate everyone else in the network as helper). The value can run from 0 to 1, ranging from nobody nominating anyone in the network as helper (value 0) to everyone nominating everyone else (value 1). An isolate is an individual that has neither outgoing nominations (outdegree = 0) nor incoming nominations (indegree = 0). The individual does not nominate helpers and is not nominated by peers as helper, and is isolated from the help network. The proportion of isolates refers to the number of isolates in a classroom relative to the size of the classroom.

**Segmentation.** A segmented network is divided into several subgroups *within which* people are closely connected to each other, and *between which* people are far removed from each other. The segmentation index [49] is based on so-called path lengths between students. Two students can be connected through a direct relation (path length 1), or indirectly through a sequence of relations (path length 2 or higher). Isolates are not connected to the network, and their path length to other students in the network is ‘infinite’. The definition of segmentation implies that there should be relatively few intermediate path lengths (path length 2) connecting students with each other relative to long path lengths (path length 3 or higher). The segmentation index compares the frequency of path lengths ≥ 2 to path lengths ≥ 3. The index can run from 0 to 1, where a network with value 1 refers to a highly segmented network in which there are no path lengths 2: it is a network of disconnected cliques. Value 0 refers to networks where there are no path lengths 3 or longer, and where all individuals in the network are connected through either directly or only one intermediary. We chose 3 as a cut-off point, as path lengths of 3 and longer are relatively less common in our data, and thus relatively lengthy, as compared to path lengths of 2. To calculate the segmentation index, we did not take into account the direction of ties. If the direction of ties were to be taken into account, there would be no path between A and C if A→B and B←C, whereas there would be if A→B and B→C. As a result, many path lengths in the network would have been ‘infinite’. To overcome this problem, we transformed the network from a directed network into an undirected network for the calculation of segmentation, in which tie A→B or A←B transforms into A↔B.

**Inequality.** We captured inequality in the distribution of help relations by calculating the (out)degree variance, which refers to the variance between students with respect to the number of helpers they nominate [50]. A higher value for inequality indicates that there is a higher variance around the mean outdegree.

**Individual network position.** Number of helpers was measured as the sum of outgoing nominations, representing how many classmates a student nominated as helper. Isolation represents whether or not a student received and gave help. If an individual did not have any incoming and outgoing nominations (referring to is an isolate in the help network), an individual was coded 1 on this variable, and 0 otherwise. Finally, centrality is, like segmentation, based on undirected path lengths. We first divided 1 by the individual path lengths to others in the network. In this way, distance ‘infinite’ became value 0, distance 4 became 0.25, 3 became 0.33, 2 became 0.50, and 1 remained 1. For each individual, we then averaged these values. This resulted in a variable running from 0 to 1, where 0 indicates that the individual is an isolate, and 1 indicates that an individual is directly connected to everyone in the network. This index is known as the Gil-Schmidt centrality index [51].

**Control variables.** Because the network indices we take into account may interrelate with the number of students in the classroom, we control for classroom size. In addition, we control for sex to take account for the differences typically found between boys and girls regarding their academic achievement [52]. Girls were coded 0 and boys were coded 1.

### Analytical strategy

**Fig 1:**
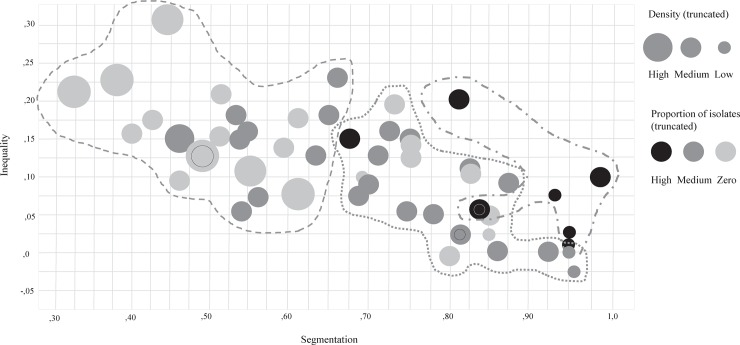
Scatterplot of the association between segmentation and inequality, with density and proportion of isolates indicated by the node’s size and color. The lines refer to the three different clusters of classrooms based on these characteristics, as identified by a K-means clustering procedure.

To describe help networks, we present the mean and standard deviation of the study variables and the correlations between them in Table 1. Following up on the correlations, in which it was seen that the classroom network structure indices correlated amongst each other, we made a scatterplot to gain better insight in the way these indices coincide in classrooms (Fig 1). Subsequently, we ran a *K*-means cluster analysis to assess whether meaningful clusters of classrooms could be identified, and presented the average academic achievement found in these clusters. The three-cluster solution is presented in Fig 1. To test our hypotheses, we employed multilevel modelling [53] using xtmixed in Stata [54]. As students were nested in classrooms (and in one school only), we distinguish two levels in our multilevel model; the individual student, and the classroom in which they are nested. We first estimated an intercept-only model in order to calculate the intraclass correlation, expressing the degree of resemblance in achievement between students residing in the same classroom. This shows how much of the variance in academic achievement can be attributed to differences between students and between classrooms. We then estimated a model with all classroom indices simultaneously, after which all individual indices were added in a second model. Because the classroom indices correlated with each other, we estimated separate models in which the individual indices and the corresponding classroom predictor were included. In addition, because individual centrality correlated highly with the other individual indices, we estimated a model without centrality. Finally, a full model was estimated in which all indices were included at once. Because no substantial differences were found as compared to the results of this full model, only the full model is presented in Table 2.

**Table 1:** Descriptives of and bivariate correlations between the study variables.

	Min.	Max.	*M*	*SD*	*N*	1.	2.	3.	4.	5.	6.	7.	8.	9.	10.
**Classroom indices**															
1.Density	.03	.29	.12	.04	54	‒	‒	‒	‒	‒	‒	‒	‒	‒	‒
2.Proportion of isolates	.00	.50	.06	.09	54	−.47**	‒	‒	‒	‒	‒	‒	‒	‒	‒
3. Segmentation	.33	.98	.69	.17	54	−.74**	.50**	‒	‒	‒	‒	‒	‒	‒	‒
4. Inequality	−.02	.30	.11	.07	54	.62**	−.23	−.67**	‒	‒	‒	‒	‒	‒	‒
5.Classroom size	12	28	21.27	4.69	54	−.20	−.43**	.02	−.09	‒	‒	‒	‒	‒	‒
**Achievement and individual indices**															
6.Academic achievement	2.97	9.37	6.88	0.93	1127	‒	‒	‒	‒	‒	‒	‒	‒	‒	‒
7.Number of helpers	0	14	2.59	2.66	1074	‒	‒	‒	‒	‒	.08**	‒	‒	‒	‒
8.% Isolation	0	1	5.40	‒	1127	‒	‒	‒	‒	‒	−.03	−.24**	‒	‒	‒
9.Centrality	0	.86	.39	.19	1074	‒	‒	‒	‒	‒	.11**	.55**	−.47**	‒	‒
10.% Sex (1 = boy)	0	1	49	‒	1138	‒	‒	‒	‒	‒	−.15**	−.26**	.13**	−.17**	‒

Note.

** p < .01.

**Table 2:** Estimated multilevel coefficients for the associations of classroom and individual network indices with academic achievement (N = 54 classrooms; 1,056 students^a^).

Parameters	Intercept only			Full model		
	*b*	*SE*	*p*	*b*	*SE*	*p*
Intercept	6.91	0.05	.000	7.84	.75	.00
**Classroom indices**						
Density				2.09	2.15	.33
Proportion of isolates				−0.13	0.78	.87
Segmentation				−0.31	0.51	.55
Inequality				−2.67	0.98	.01
Classroom size				−0.03	0.01	.02
**Individual indices**						
Number of helpers				0.00	0.01	.82
Isolation				0.11	0.14	.46
Centrality				0.51	0.29	.08
Sex				−0.29	0.06	.00
Classroom variance	.12	.03		.08	.02	
Individual variance	.74	.03		.72	.03	
Likelihood ratio test (*df* = 9)				56.3		.00

Note.

^a^ Decrease in analytical sample size because of missing values.

## Results

### Description of academic achievement

Academic achievement was normally distributed with values ranging between 2.97 and 9.37, and a mean of 6.88 (*SD* = 0.93).

### Description of classroom network indices

The density in classrooms ranged from .03 to .29, with an average of .12 (*SD* = .04). Thus, in the densest help network, about one third of all possible help relations in the classroom were actual relations, whereas this was 12% on average. The proportion of isolates ranged from 0 to .50 (*M* = .06, *SD* = .09), where .50 was an outlier (it was followed by value .31). If there were any isolates in the classroom, their number ranged from 7 (followed by 4) to 1. In many networks (22 of the 54 classrooms), however, no one was excluded from help relations.

**Fig 2:**
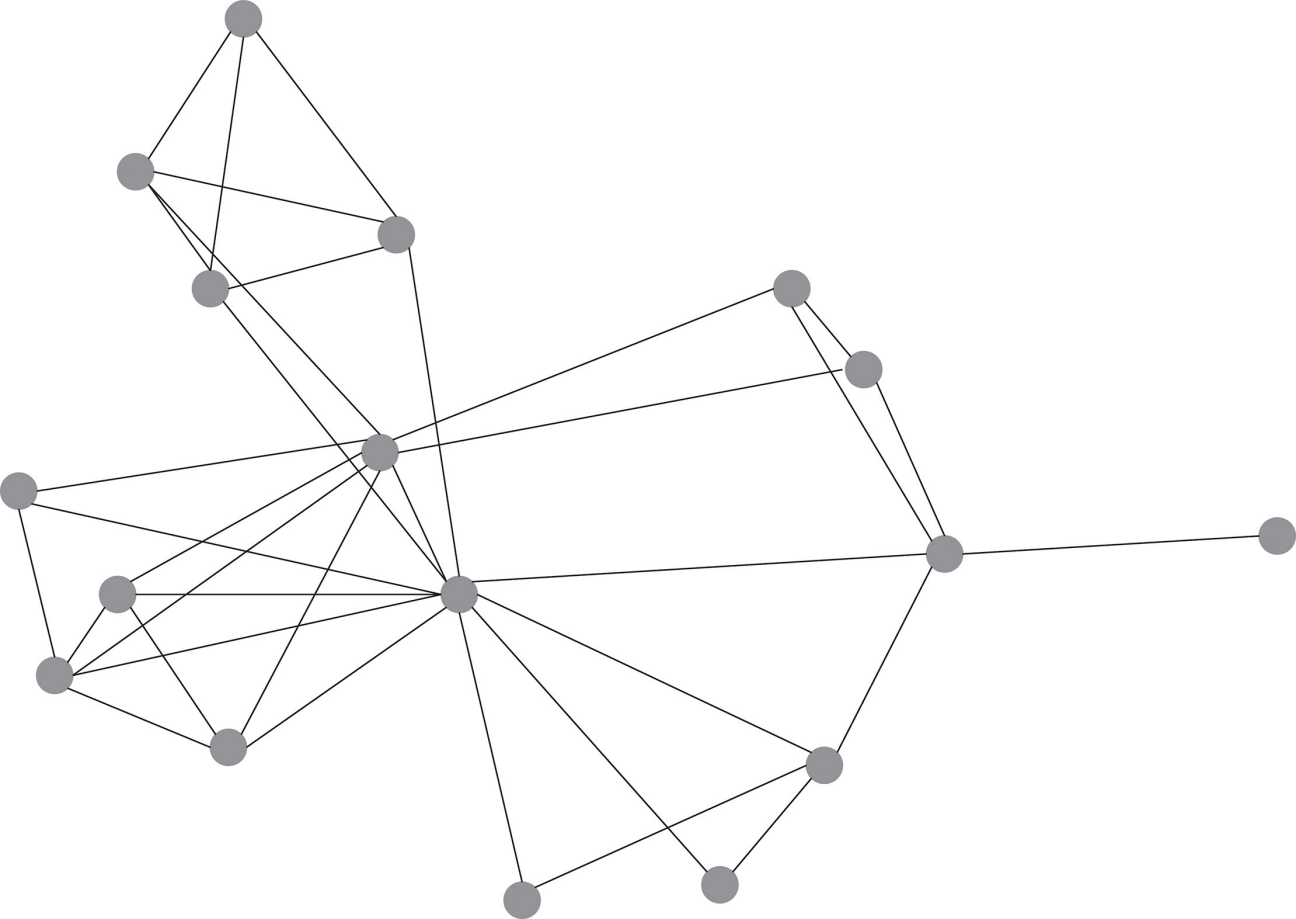
Sociogram of a help network with low segmentation (value .33) and high inequality (value .21).

**Fig 3:**
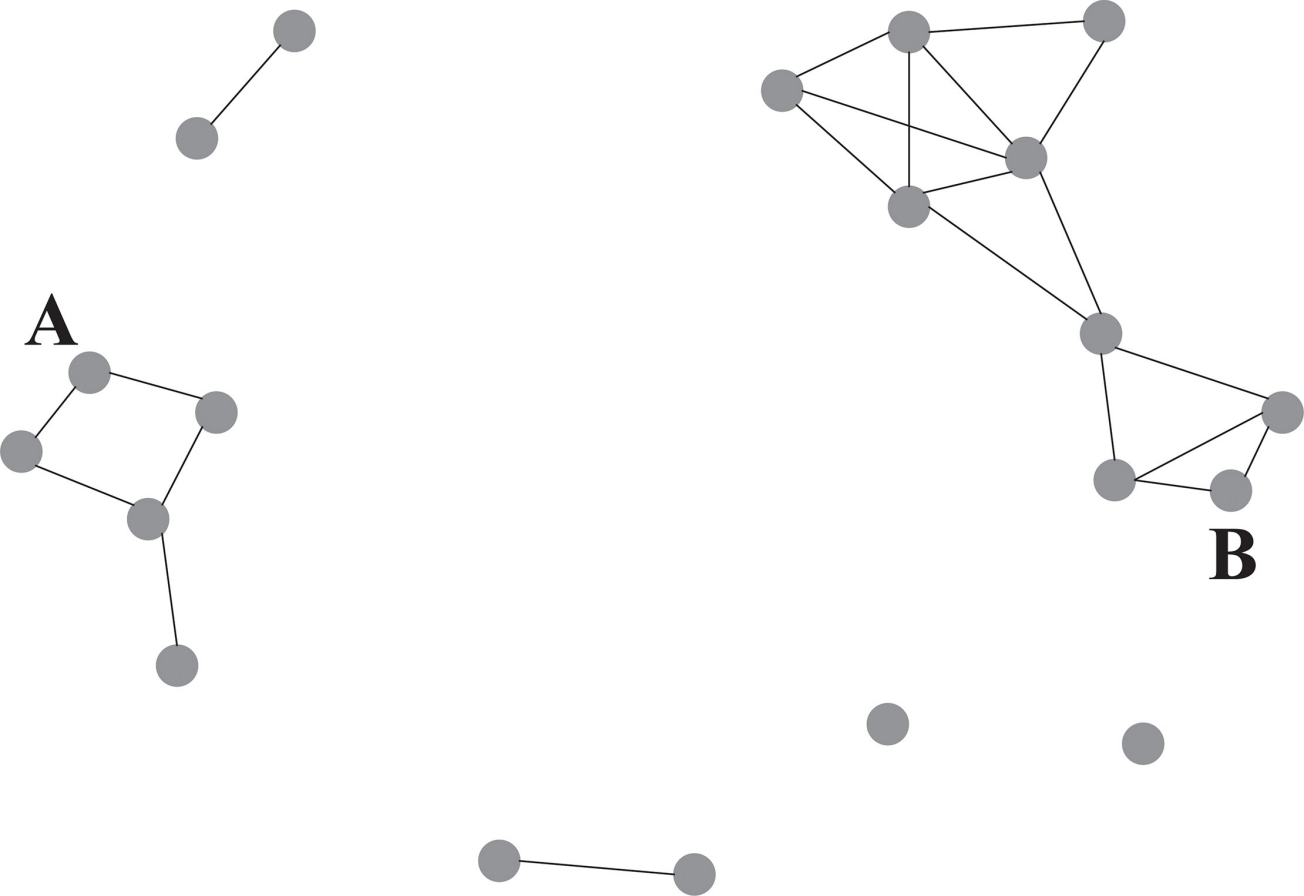
Sociogram of a help network with high segmentation (value .92) and low inequality (value .10), highlighting individuals with relatively low (A) and high (B) centrality.

The segmentation index ranged from .33 to .98, with an average of .69 (*SD* = .17). This means that every classroom was segmented to some extent. Even in the classroom with the lowest segmentation index, 50% of the path lengths was intermediate (distance 2), and 50% of the path lengths was short (distance 1) or relatively long (distance 3 or 4). In the classroom with the highest segmentation index, only 2% of the path lengths was distance 2, and 98% of the paths lengths was distance 1 or infinite. Differences in segmentation are illustrated by the sociograms depicted in Fig 2 and Fig 3, in which the nodes represent individuals and the lines represent a help relation. The classrooms in Fig 2 and Fig 3 scored low (.33) and high (.92), respectively, on the segmentation index.

Inequality ranged from −.02 to .30 (*M* = .11, *SD* = .07). In the classroom with the lowest inequality, each individual had one or two helpers, and one individual mentioned three. In the classroom with the highest inequality, the number of helpers generally ranged from 1 to 3, but three individuals mentioned 4, 7, and 9 helpers. Illustrating the direction of inequality, we found that the skewness of the individual number of helpers was positive in all classrooms (*M* = 1.09, *SD* = 0.53, *min* = 0.07, *max* = 2.17). This indicates that, generally, most students had a few helpers, whereas some had many helpers. In the classrooms with a moderate positive skew, the number of helpers was more evenly spread over the range of number of nominations in that classroom. The classrooms in Figs 2 and 3 scored high (.21) and low (.10), respectively, on inequality.

In sum, most students were connected to classmates through help relations. However, students tend to cluster in help cliques, making the networks generally segmented. Lastly, in most classrooms, there was a tendency for help relations to be unequally distributed over students. This was caused by the presence of some students who were helped by relatively many others. In relatively equal help networks, this was less the case.

### Description of individual position in the help network

On average, students mentioned about 2.59 classmates as helper (*SD* = 2.66). Some students nominated no helpers, whereas others nominated as many as 14. There were 61 isolates in the sample (5.4%), spread out over 32 classrooms. Individual centrality scores varied widely over individuals, with some students being isolates (value 0), and some individuals very well positioned in the network (value .86). The number of help relations students had, however, did not necessarily improve their centrality in the network. In Fig 3, for example, individuals A and B had the same number of relations (three), but individual B (centrality score .26) was better positioned than individual A (centrality score .18), as B may access more classmates in the network through relatively short path distances.

### Correlations

Bivariate correlations between the network indices are displayed in Table 1. Density was moderately and negatively correlated with the proportion of isolates (*r* = −.47, *p* < .01), positively with inequality (*r* = .62, *p* < .01), and more strongly and negatively with segmentation (*r* = −.74, *p* < .01). Segmentation was moderately and positively related to the proportion of isolates (*r* = .50, *p* < .01) and more strongly and negatively related to inequality (*r* = −.67, *p* < .01). Regarding individual network position, especially centrality was correlated with other individual variables: Centrally positioned individuals reported a large number of helpers (*r* = .55, *p* < .01) and were less isolated (*r* = −.47, *p* < .01). Achievement was positively correlated with the number of helpers and centrality (*r* = .08, *p* < .01; *r* = .11, *p <* .01), although these correlations were small. Finally, boys had lower school grades than girls (*r* = −.15, *p* < . 01).

### Further exploration of the correlations: Scatterplot of classroom network indices and *K*-means cluster analysis

To gain better insight into the correlations of classroom network indices amongst each other, we produced a scatterplot in which classrooms are represented by nodes (Fig 1). The position of the nodes on the X-axis and Y-axed was determined by their value for segmentation and inequality. Each node has a color (black, dark grey, or light grey), corresponding to the level of network density. For the purpose of clarity, density was truncated into low, medium, and high values (lower than, around one, and higher than one standard deviation from the mean density). The nodes also differ in size, corresponding to low, medium, or high values of the proportion of isolates. The proportion of isolates was also truncated (no isolates, up to one standard deviation from the mean, and higher than one standard deviation from the mean).

First, as the negative correlation implies, the scatterplot indicates that there was little inequality in highly segmented classrooms. However, we expected both to be indicators of positive classroom atmospheres, and thus, implicitly, that these would correlate positively. Further inspection of the networks revealed that this negative correlation was caused by a few individuals that were highly central in the network (increasing inequality), who, at the same time, linked classmates from different help cliques together (lowering segmentation). The presence of these central individuals also explains the positive link between density and inequality. Thus, the inequality in the network seemed to counter segmentation of the network, and heightened network density. Second, the scatterplot demonstrates that classrooms theoretically most beneficial for achievement (with an equal distribution of nominations, little segmentation, a few isolates, and / or high density), of which some can be found in the lower left section of the plot, were highly uncommon in our sample. Thus, there were hardly any classrooms in which the positive network characteristics all coincide. Lastly, the scatterplot shows that it is difficult to identify a typical classroom, as classrooms vary widely in their network structure. Only a few classrooms looked similar on all four dimensions (e.g., the three top left classrooms all have comparable segmentation, inequality, density, and proportion of isolates). However, in an attempt to typify classrooms based on their network characteristics, we ran a K-means cluster analysis using density, the proportion of isolates, inequality and segmentation as input variables. The algorithm of the *K*-means cluster analysis aims to minimize within-cluster heterogeneity, and can as such identify groups of classrooms that are relatively similar on the four network dimensions. The algorithm does not identify the number of clusters, and these have to be specified by the user. In our case, the three-cluster solution seemed most optimal, as the number of classrooms in each cluster became very small starting from a 5-cluster solution, and the 4-cluster solution identified clusters of which two were quite similar on all dimensions.

The first, most typical group of classrooms (*N* = 26) is depicted on the right side of the scatterplot, and was characterized by medium density (*r* mean = .11), low proportion of isolates (*r* mean = .05), low inequality (*r* mean = .08) and high segmentation (*r* mean = .79). Theoretically, this type of classroom seems beneficial for achievement, although it is characterized by high segmentation. The second cluster (*N* = 22) perhaps theoretically most beneficial for achievement, is depicted on the left side of the scatterplot, and was characterized by high density (*r* mean = .15), a few isolates (*r* mean = .02), and medium levels of inequality and segmentation (*r* mean = .16; .52). The third and smallest cluster (*N* = 6) was primarily characterized by a high proportion of isolates (*r* mean = .27). This cluster also showed medium density (*r* mean = .07), low inequality (*r* mean = .08) and high segmentation (*r* mean = .91). Although it is difficult to statistically compare means based on a relatively small number of classrooms, the clusters showed comparable average achievement scores, with the highest achievement scores in the second cluster (cluster 1: *M* = 6.84, *SD* = 0.35; cluster 2: *M* = 7.01, *SD* = 0.41; cluster 3: *M* = 6.89, *SD* = 0.58).

### Results multilevel analysis

For each variable, Table 2 presents the estimated coefficients *b*, their standard errors *SE*, and the *p*-values for testing the value of 0. We present the likelihood ratio test, comparing the fit of the full model as compared to the intercept-only model. The intercept-only model is presented in the first column of Table 2, and was used to estimate the intraclass correlation. Results suggested that 14% of the variance in academic achievement could be attributed to differences between classrooms, and 86% could be attributed to differences between students. The full model was a significant improvement of the intercept-only model (*χ*^*2*^ = 56.3, *df* = 9, *p* < .001), and explained 33.3% of the variance on the classroom level and 3.7% of the variance on the individual level. For interpreting the effects on achievement, note that achievement grades ranges from 3.0 to 9.4, with a standard deviation of 0.9.

At the classroom level, the results reveal that inequality was significantly associated with achievement (*b* = −2.67, *SE* = 0.98, *p* = .01), which is consistent with our hypothesis that achievement was lower in more unequal classroom help networks (Hypothesis 3). The result indicates that achievement decreased by about one point if inequality increased by one third of the range of inequality. Hypothesis 1 and 2 were rejected, as there were no associations of density, proportion of isolates, or segmentation with achievement. Classroom size had a small, but significant negative association with achievement (*b* = −0.03, *SE* = 0.01, *p* = .02), indicating that achievement decreased by 0.03 points when classroom size increased with one individual. Decreasing 0.5 point in academic achievement would thus ‘require’ an increase of 17 in classroom size, demonstrating that the association is relatively small.

At the individual level, the model suggests that individual centrality in the help network was positively associated with achievement although the effect was small (*b* = 0.51, *SE* = 0.29, *p* = .08). Thus, the hypothesis stating that centrally positioned individuals show higher achievement (Hypothesis 6) had no strong support. Hypothesis 4 and 5 were rejected, as individual number of helpers and isolation were not associated with achievement. Lastly, and as expected, boys had lower academic achievement than girls (*b* = −0.29, *SE* = 0.06, *p* < .001).

**Supplementary analyses.** Because associations of classroom and individual network indices with achievement may vary between classrooms, we tested for random slopes. Furthermore, we tested for interactions between the classroom network indices and individual network position, as effects of the classroom network may depend on one’s individual network position. For example, a highly dense help network might not be beneficial for individuals with a few helpers. In addition, as help is more salient for girls’ than for boys’ relationships [55–57], we tested for interactions between sex and the network and individual indices. None of the random slopes or interactions were significant.

## Discussion

This study investigated the structure of adolescent classroom help networks, the positions individuals take up in this help network, and their associations with academic achievement. We captured help networks by asking adolescents to mention classmates who ‘*help them with problems (for example*, *with homework*, *with repairing a flat [bicycle] tire*, *or when you are feeling down)*?’ Aggregating these help relations to the classroom level demonstrates how students in a classroom are precisely connected with each other through help. We assessed the extent to which help relations were present (cohesion), segmented (clustered in groups), and unequally distributed over students (inequality). Similarly, on the individual level, we assessed how many helpers students had, whether students were isolates (referring to that they did not give or receive help at all), and the centrality of their position in the help network. Subsequently, we examined how these network indices were associated with adolescents’ academic achievement.

### Classroom help networks and individual network positions

To our knowledge, classroom help networks in adolescence have not been investigated, which is why we looked further into how help networks look like within and across classrooms. First, whereas part of the aim of this study was to provide a coherent description of what classroom help networks generally look like, they appeared challenging to characterize; they varied in density, proportion of isolates, segmentation, and inequality, and there were only a few classrooms that were similar in these four dimensions. This finding underlines the complexity of the social context in which students and teachers spend their days, and that it may be difficult to speak of a typical classroom, or a classroom that is typically ‘good’ or ‘bad’ for adjustment. For educational practice, the intricacy of this social context suggests that designing school-wide interventions aimed at improving student well-being is challenging.

Second, we found that classroom help networks that were theoretically expected to be most beneficial for academic success (referring to classrooms with high density, a few isolates, little segmentation, and low inequality) were highly uncommon in our sample. In addition, every classroom help network was segmented and unequal to some extent. The absence of ‘ideal’ classrooms might suggest that researchers should adjust their notion of what is a beneficial or detrimental social environment for adolescents. Students organize themselves in ways that are theoretically speaking not ideal for their adjustment; apparently, this is the natural way in which they organize their social environment. This stresses a need to delineate the mechanisms underpinning the complex and theoretically ‘counterintuitive’ structure of social relations. Possibly, ‘suboptimal’ network structures may arise through the self-organizing capacity of networks [58]: preferences for relationship formation at the individual level (such as the tendency to reciprocate help, or help similar others [47]) may have unintendedly contributed to the segmented network structure found at the classroom level. These seemingly universal principles may nonetheless result in diverging classroom network patterns–as our results demonstrated, not all classrooms are segregated into groups of similar peers to the same extent. Network ecology theory [59] emphasizes that features of the classroom context may amplify or attenuate preferences for relationship formation and contribute to variation in characteristics of the larger network. In line with this, individual tendencies toward nominating others as helper, reciprocating help nominations and nominating helpers-of-helpers as own helper vary over contexts [47]. Heterogeneity in the characteristics of students in a classroom is a contextual characteristic that pertains to variation in trust and openness, and may thus be relevant for explaining variation in help networks [59]. It is argued that heterogeneity increases the opportunity for social segregation; the more common a certain attribute is (e.g., some classrooms were characterized by high percentages of Dutch or female students) the less relevant this attribute becomes for social selection. More importantly for help, however, is that heterogeneity of attributes in the social context may raise concerns and uncertainty about others’ trustworthiness [59, 60], amplifying the tendency to limit help interactions with others with whom one can more readily identify similar others [14]. Thus, heterogeneity may reduce feelings of trust and openness, resulting in segregated and possibly low-density help networks in which peers establish help relations with a selective set of similar classmates.

This finding also emphasizes a need to examine in more detail in what way certain characteristics of social networks are beneficial or detrimental for adjustment. For example, inequality might not be detrimental to the classroom atmosphere if the individuals that report relatively many helpers are indeed in high need of help; or isolation of individuals in the help network may be detrimental to achievement only if these individuals are isolated from other positive networks as well. The finding that students organize their help networks in a way that is theoretically ‘suboptimal’, but otherwise natural also raises the question whether teacher intervention in these naturally emerging social settings would eventually affect students’ adjustment in a favorable way. Research suggested that teachers’ attempt to foster social relations through grouping arrangements may result in adverse classroom outcomes, such as a higher acceptance of aggressive behaviors and lower acceptance of prosocial behaviors [61]. Perhaps, social network information might be of use only if teachers cooperate with students in interpreting this information: Teachers may discuss the classroom social network and social network positions together with their students, so that changes in social relations are in accordance with students’ preferences.

Although classrooms generally showed ‘suboptimal’ help network patterns, students’ individual network positions seemed to be more in line with what is considered beneficial for adjustment. Most individuals indicated that they received help from their classmates, and there were hardly individuals that were completely isolated from the helping network. These descriptive findings reveal an interesting discrepancy: On the individual level, help seems optimally organized, yet aggregating help relations to the classroom level shows how a potential suboptimal social process (referring to inequality) is taking place and affects achievement. This underlines the importance of taking into account individual embeddedness in a social context together with characteristics of this social context when investigating adolescent adjustment, or developing interventions aimed at improving adolescents’ adjustment.

### Associations of classroom help network characteristics with achievement

Building on earlier research [12], we generally expected students’ academic achievement to flourish in classrooms in which many students helped each other, and in which help relations were equally distributed and not segmented. Our multilevel results were partly consistent with the expectations. It was shown that higher inequality in the distribution of helpers was associated with lower academic achievement. This finding was in line with previous findings on inequality in social relations and behavioral outcomes [26] and it was argued in these studies that inequality may affect outcomes, such as achievement, by triggering feelings of comparison and competition amongst students [4]. In our study, inequality was high in classrooms where there were a few central students who reported to have many helpers among their classmates, whereas there were many students who reported only a few helpers. This type of inequality has been often found in social networks [62, 63] and has been referred to as a process of preferential attachment: individuals who have many social relations tend to attract additional social relations in the future. Although we assessed help networks only at one time point, it might have been that students who received help from many classmates at some point received even more help at later time points (a ‘rich- get-richer’-effect). Thus, over time, access to helpers (and to their skills and knowledge) might have concentrated on a relatively small set of classmates. This may trigger not only competition among students, but also feelings of injustice; students may find a situation in which only some central classmates benefit from the skills and knowledge of classmates unfair. These mechanisms, argued to hamper achievement by undermining a positive classroom atmosphere, have not been tested. More research is needed about the mechanisms underpinning the consistent negative association found between inequality and adolescent adjustment.

Although we expected that classroom segmentation would be associated with lower achievement, we did not find support for this expectation. We argued that positive traits that accompany giving and receiving help, such as respect and trust, would be more widespread in classrooms where students helped peers also outside the boundaries of specific groups of classmates [64]. This would positively affect achievement. Given that we found no association between segmentation and achievement, it might be that segmentation might be detrimental to achievement only if students are not only structurally, but also socially segmented; if subgroups emerge of students similar on, for example, skills. Similar students in subgroups may be less suitable to help each other, as they likely seek solutions for similar problems. For example, students in subgroups of lower achievers may all need help with mathematics. Contact with dissimilar others may, however, bring them in contact with peers having complementary characteristics (e.g., high achievement). These peers may, through their complementarity, be better able to tackle their problems. As such, a less socially segmented classroom help network may more easily bring help seekers in contact with suitable help providers. However, in contrast, some research suggests that achievement flourishes in classrooms where students tend to hang out with a specific set of classmates [27]. Perhaps, in segmented classrooms in which students focus only on their own group of (similar) helpers, students develop more high- quality help relationships in which respect and trust are more deeply engrained. This may benefit achievement more than having superficial help relationships with many classmates. Future research may provide more insight into these contrasting findings, and investigate in more detail in what way segmentation may hamper or contribute to students’ achievement.

Contrary to our expectations, we did not find an association between the proportion of isolates, referring to students in the classroom that were not involved in giving and receiving help, and achievement. Our expectation followed from previous empirical findings, showing that marginalization of students in the classroom, specifically bullying, also affected the wellbeing of other students in the classroom, irrespective of whether these students were bullied themselves [22–24]. It might be that marginalization from positive social relations, such as help, is less notable than marginalization in a negative network, such as bullying–bullying arguably is more visible behavior that might affect feelings of safety of all. In addition, statistically speaking, effects of isolation were challenging to detect, as there were not many students who were isolated from the helping network.

Taken together, only inequality in the helping network was associated with academic achievement. Future research should assess in more detail why inequality in social relations is so consistently related to adverse student outcomes across studies; and should assess in more detail how cohesion and segmentation in social networks is associated with adjustment. In addition, we assumed that cohesion, equality, and low segmentation were all reflective of a positive classroom social climate, fostering academic achievement. However, the indices were not consistently related to achievement, stressing the need to examine what structure of social relations constitutes a beneficial social climate; and which individual and contextual processes precede the classroom social structure.

For educational practice, the finding that inequality hampers achievement would imply that teachers should intervene in a classroom in such a way that help relations become more equally distributed. Perhaps, this idea can be integrated in peer tutoring interventions in which students are grouped together to mentor each other in their school motivation and achievement [65, 66].

### Associations of individual position in the help network with achievement

Apart from characteristics of the classroom help network, we investigated associations between individual position in the help network and achievement. Particularly, we hypothesized that achievement was higher for individuals that were in the position to reach many helpers–not only directly, but also through indirect help relations with classmates [7, 38, 45].

Our results suggested that the number of helpers and isolation from the help network did not relate to achievement. By how many peers students were helped directly was unrelated to achievement, but centrality seemed to be positively associated with achievement. In other words, it might matter whether students can easily access potential helpers in the classroom through other classmates. This might reflect indirect help relations as instrumental for reaching academic goals; direct help relations might be more intimate, and may matter more for mental well-being instead of achievement [45].

Whereas null findings regarding individual network position contrasted with our expectations, there is some research that ties in with these results: For example, no association was found between the number of peers students mentioned as academic advisor and achievement [67]. What is more, ‘neglected’ students, referring to students who were not involved in positive nor negative social relations in the classroom, performed quite well in school [40]. The absence of associations between individual network position and achievement suggests that other individual characteristics, such as academic self-efficacy, might be more salient for the prediction of achievement than students’ individual help connections.

### Limitations and further research

The following limitations to this study should be taken into account when interpreting the results. First, we assessed the association of help in a broad sense (referring to help with homework, with repairing a flat tire, or when feeling down) with achievement in a narrow sense (referring to school grades). A social network structure assessed with peer nominations for more specific instances of help, for example, help with school assignments, would have likely related more clearly to academic achievement. Our measure of help aligned with our aim to capture the general tendency of students to help each other, and herewith a supportive classroom climate. Also, our broad definition of help, suggesting that everybody once in a while will need some help, aimed to minimize the role that the need for and ability to help may have otherwise played in explaining the structure of the help network.

Second, we could not study the influence of network structure and individual network position on academic achievement in a longitudinal framework. Individual social relationships are subject to change, and may as such change the position of individuals, and the structure of the network as a whole. Previous research among early adolescents demonstrated that there is turnover in whom individuals mention as best friends at the beginning and end of already a three-week period [68]. The turnover in help, showing less stability than friendships [47] might be even higher. Therefore, to obtain a more detailed view of the co-evolution of networks and (school) outcomes, future research should consider including multiple measurements with short-term intervals of networks and focal outcomes.

The focus of this study was on the classroom social climate as captured by peer relations. The teacher, however, plays a significant role in shaping the classroom climate and academic achievement as well [3, 25]. Future research should get more insight in the feedback processes between teachers, the structure of classroom social networks, and academic achievement. For example, features of networks may improve teachers’ ability to teach or manage the classroom, or particular teaching practices may alter the structure of the network, which may affect achievement.

Although the strength of this paper is that we looked explicitly at help relations to capture a supportive classroom climate, these help relations overlap with other social relationships, such as friendship, that contribute to the classroom social climate [69, 70]. Future studies might further investigate the extent to which classroom level network patterns overlap, and investigate how the dynamics of multiple networks shape the classroom social climate, and affect adjustment. For example, it might be examined whether students limit the exchange of help to friends or whether help extends beyond the borders of friendship, and whether this affects school and other outcomes.

## Conclusions

This study has moved the field on classroom climate forward by being the first to examine the structure of classroom help networks, individual positions in these networks, and their associations with achievement in a large sample of adolescents. At the classroom level as well as individual level, the number of help relations did not affect achievement. Also, it did not matter for achievement whether students limit their help interactions to a specific set of peers. Of importance for academic success was that help relations in the classroom are equally distributed, and, potentially, that individuals can easily access classmates for help. The division of classrooms into ‘haves’ and ‘have-nots’ in terms of access to help may lead to feelings of injustice or competition between students, undermining a positive classroom atmosphere, and hampering achievement.
